# Supplementary material for: Evaluating topological variability in Neodermata phylogenies using mitochondrial and ribosomal gene markers
Source: PLoS One. 2025 Oct 15;20(10):e0333282. doi: 10.1371/journal.pone.0333282 (PMC12527160; doi:10.1371/journal.pone.0333282)
Supplement: S1 Fig — Two species of planarians (Schmidtea mediterranea and Macrostomum lignano) were used as an outgroup, using three different software. Ninety unilocus trees and six multilocus trees were constructed using nucleotide and amino acid sequences derived from mitochondrial genes. Furthermore, three multilocus trees were inferred using the nucleotide sequences of mitochondrial genes + 18S NrRNA + 28S NrRNA, three multilocus trees using the nucleotide sequences 16S MrRNA + 18S NrRNA + 28S NrRNA + cytb, and six unilocus trees using NrRNA. (PDF) [file pone.0333282.s001.pdf]

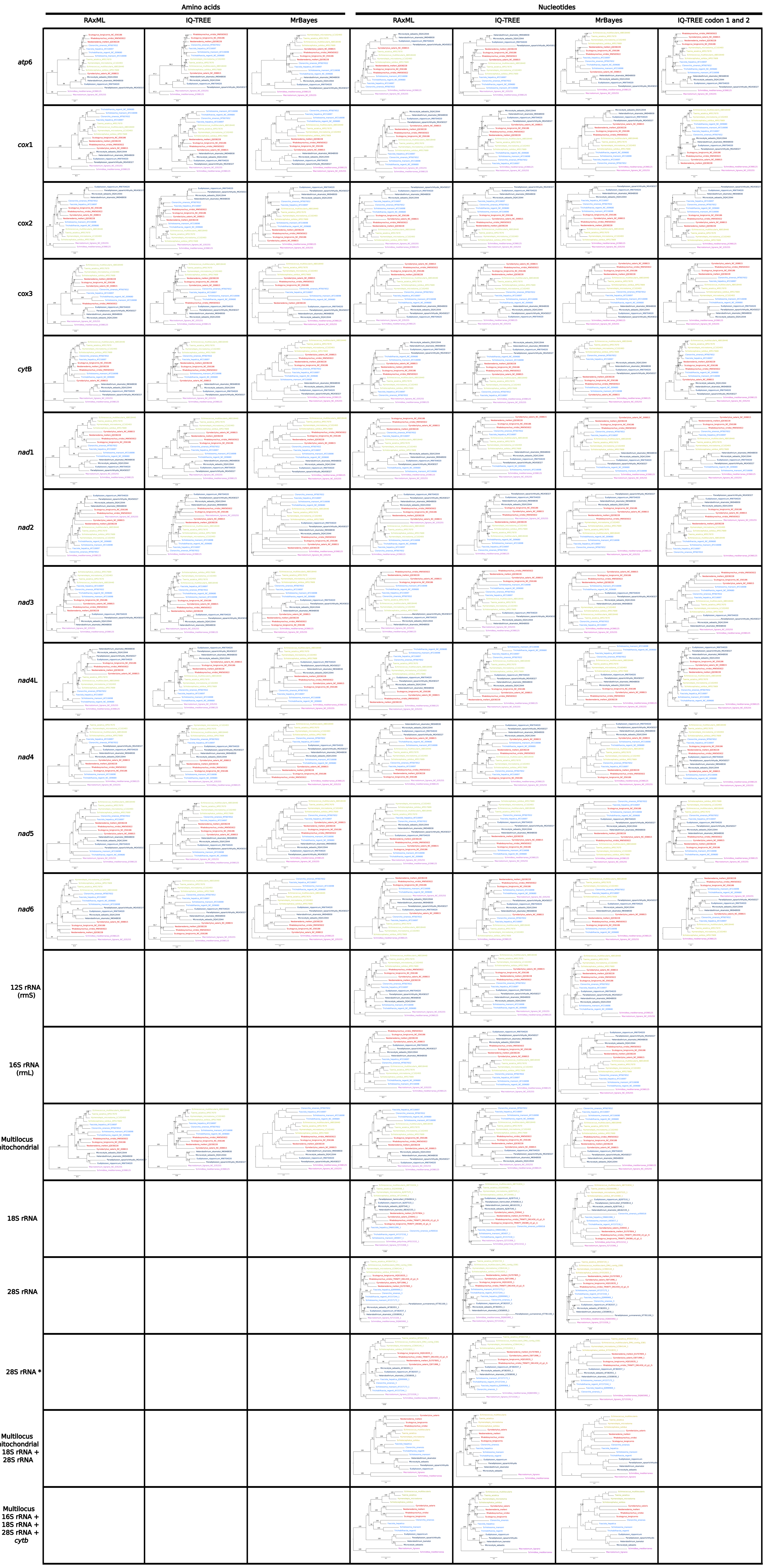

Supplementary Figure S1. Phylogenetic trees obtained from mitochondrial and nuclear genes of 16 species of the Neodermata, and two species of planarians (*Schmidtea mediterranea and Macrostomum lignano*) as an outgroup, using three different software. Ninety unilocus trees and six multilocus trees were constructed using nucleotide and amino acid sequences derived from mitochondrial genes. Furthermore, three multilocus trees were inferred using the nucleotide sequences of mitochondrial genes + 18S NrRNA + 28S NrRNA, three multilocus trees using the nucleotide sequences 16S MrRNA + 18S NrRNA + 28S NrRNA + *cytb*, and six unilocus trees using NrRNA.
