# Supplementary material for: Evaluating topological variability in Neodermata phylogenies using mitochondrial and ribosomal gene markers
Source: PLoS One. 2025 Oct 15;20(10):e0333282. doi: 10.1371/journal.pone.0333282 (PMC12527160; doi:10.1371/journal.pone.0333282)
Supplement: Attachment — Submitted filename: Response to Reviewers.docx [file pone.0333282.s012.docx]

Manuscript PONE-D-25-05422R1

Although it is my view that most of the concerns that reviewer #2 has raised can be classified as concerns about the scientific importance of your work (which is not a criteria PLOS ONE uses to assess the suitability of manuscripts), they have identified a concern about the article´s intelligibility which must be addressed before the work can be published in PLOS ONE. Specifically, the referee has highlighted that there is no mention, in the results or discussion, of the “silhouette widths” we are told you have calculated in your methods section. I am, therefore, requesting that you modify your manuscript to either exclude mention of these calculations in the methods or mention the measurements in the results and discussion (and expect a further round of peer-review).

Done. We have removed mention of silhouette width analysis and reference 35 which mentioned the package used.

Please also correct the following typographical errors:

Abstract (line 33): Please change “three software” to “three software packages”.

Done.

Discussion (line 283): Please change “uur objective” to “our objective”.

Done.
